# Medical College Consolidation

**Published:** 1899-04

**Authors:** 


### Medical College Consolidation.

The era of mushroom medical schools seems to be drawing to a close, and that of consolidation of existing schools to have begun. We have noted the union of the University of Bellevue in New York and of two colleges in Atlanta, and now it is announced that the medical department of Niagara University has been consolidated with the medical department of the University of Buffalo. This a healthy movement, which we hope, in the interest of sound medical education, will not be checked until it has spread over the entire land.—*N. Y. Med. Record.*

